# Factors Contributing to Surgical Site Infections: A Comprehensive Systematic Review of Etiology and Risk Factors

**DOI:** 10.3390/clinpract14010006

**Published:** 2023-12-28

**Authors:** Alexandra Bucataru, Maria Balasoiu, Alice Elena Ghenea, Ovidiu Mircea Zlatian, Dan Dumitru Vulcanescu, Florin George Horhat, Iulia Cristina Bagiu, Virgiliu Bogdan Sorop, Madalina Ioana Sorop, Andrada Oprisoni, Estera Boeriu, Stelian Stefanita Mogoanta

**Affiliations:** 1 Doctoral School Department, University of Medicine and Pharmacy of Craiova, 200349 Craiova, Romania; alexandra.catana95@gmail.com; 2 Infectious Disease Department, Victor Babes University Hospital Craiova, 200515 Craiova, Romania; 3 Department of Bacteriology-Virology-Parasitology, University of Medicine and Pharmacy of Craiova, 200349 Craiova, Romania; maria.balasoiu@umfcv.ro (M.B.); alice.ghenea@umfcv.ro (A.E.G.); ovidiu.zlatian@umfcv.ro (O.M.Z.); 4 Department of Microbiology, “Victor Babes” University of Medicine and Pharmacy, Eftimie Murgu Square 2, 300041 Timisoara, Romania; dan.vulcanescu@umft.ro (D.D.V.); horhat.florin@umft.ro (F.G.H.); 5 Multidisciplinary Research Center on Antimicrobial Resistance (MULTI-REZ), Microbiology Department, “Victor Babes” University of Medicine and Pharmacy, Eftimie Murgu Square 2, 300041 Timisoara, Romania; 6 Department of Obstetrics and Gynecology, “Victor Babes” University of Medicine and Pharmacy, Eftimie Murgu Square, No. 2, 300041 Timisoara, Romania; bogdan.sorop@gmail.com; 7 Doctoral School, “Victor Babes” University of Medicine and Pharmacy, 300041 Timisoara, Romania; pop_madalina_91@yahoo.ro; 8 Department of Pediatrics, Discipline of Pediatric Oncology and Hematology, “Victor Babes” University of Medicine and Pharmacy, Eftimie Murgu Square, No. 2, 300041 Timisoara, Romania; oprisoni.licinia@umft.ro (A.O.); estera.boeriu@umft.ro (E.B.); 9 Third General Surgery Department, Clinical Emergency County Hospital, 200642 Craiova, Romania; stelian.mogoanta@umfcv.ro; 10 Department of General Surgery, Faculty of Dental Medicine, University of Medicine and Pharmacy of Craiova, 200349 Craiova, Romania

**Keywords:** surgical site infections (SSIs), antimicrobial resistance, risk factors

## Abstract

Surgical site infections persist as a substantial concern within the realm of hospital-acquired infections. This enduring issue is further compounded by the mounting challenge of antibiotic resistance, a surge in surgical interventions, and the presence of comorbidities among patients. Thus, a comprehensive exploration of all discernible risk factors, as well as proactive preventive and prophylactic strategies, becomes imperative. Moreover, the prevalence of multidrug-resistant microorganisms has reached alarming proportions. Consequently, there is an acute need to investigate and scrutinize all potential therapeutic interventions to counter this burgeoning threat. Consequently, the primary objective of this review is to meticulously assess the origins and risk elements intertwined with surgical site infections across a diverse spectrum of surgical procedures. As the medical landscape continues to evolve, this critical analysis seeks to provide a nuanced understanding of the multi-faceted factors contributing to surgical site infections, with the overarching aim of facilitating more effective management and mitigation strategies. By exploring these dimensions comprehensively, we endeavor to enhance patient safety and the quality of surgical care in this era of evolving healthcare challenges.

## 1. Introduction

### 1.1. Background

Surgical site infections (SSIs) stand as a prominent category within the realm of healthcare-associated infections, emerging within a span of 30 days following a surgical procedure at the precise site or anatomical region where the surgery unfolded [1]. These infections, though challenging, are comprehensively understood through a multifaceted classification that guides healthcare professionals in their prevention and management efforts.

The Centers for Disease Control and Prevention (CDC) delineates surgical wounds into four distinct categories, each defining a unique level of risk and infection potential [1]. Clean wounds, characterized by their uninfected state and absence of inflammation, necessitate meticulous attention, especially during drainage procedures. Moving forward, clean-contaminated wounds traverse the respiratory, gastrointestinal, or genitourinary tracts under meticulously controlled aseptic conditions. Although a minimal risk of contamination is acknowledged, infections remain rare. Contaminated wounds, juxtaposed, present as open wounds marked by minor lapses in sterility or incisions that exhibit acute or aseptic inflammation. Finally, the realm of dirty or infected wounds encompasses traumas inadequately treated, featuring pre-existing infection or foreign material contamination at the surgical site.

Another avenue of classification stems from the depth of infections, subdividing SSIs into three categories: superficial SSIs, deep incisional SSIs, and organ space SSIs (Table 1) [1]. Superficial SSIs confine their presence to the skin or subcutaneous tissue, manifesting within the initial 30 days post-surgery. Deep incisional SSIs traverse deeper layers, including fascia and muscle, emerging within 30 days or up to a year if an implant is in situ. At the pinnacle of severity, organ space SSIs beckon our attention with heightened morbidity and mortality risks. This dire manifestation surfaces within 30 days or one year (in cases involving implants) and involves the abdominal cavity, intricately manipulated during the surgical maneuver [2].

**Figure 1:**
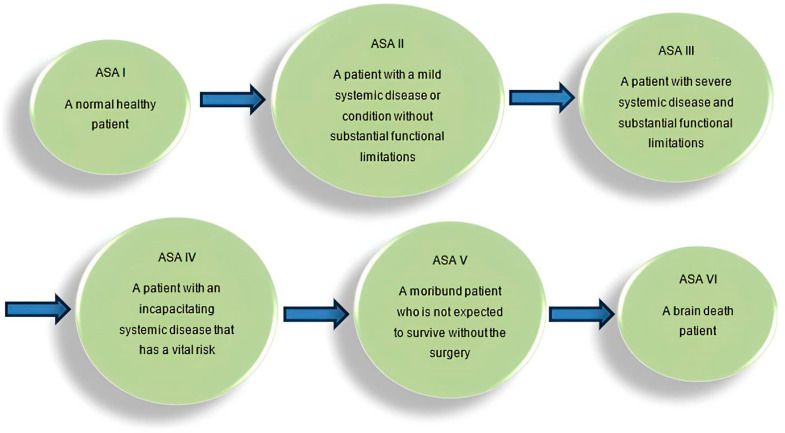
Operative risk assessment flowchart.

In a concerted effort to provide clinicians with a clear snapshot of a patient’s physio-logical status, the American Society of Anesthesiologists (ASA) devised a concise physical status classification system [3]. This strategic system aids in estimating operative risk, con-tributing to better-informed decisions throughout the perioperative journey (Figure 1).

Amid the landscape of healthcare-associated infections (HAIs), SSIs hold substantial sway, underpinning a substantial portion of morbidity, extended hospital stays, and augmented healthcare expenditures. The CDC underscores the prominence of SSIs, attributing approximately 20% of HAIs to this category alone. In terms of fiscal impact, SSIs command the highest economic toll, tallying an annual average cost of USD 3.3 billion [4].

In the year 2017, the European Centre for Disease Prevention and Control (ECDC) documented a total of 10,149 SSIs, offering a comprehensive glimpse into the prevalence and distribution of these infections [4]. The spectrum of SSIs unraveled as follows: 47% (4739 cases) encompassed superficial SSIs, 30% (3088 cases) fell under the ambit of deep SSIs, while 22% (2274 cases) materialized as organ/space SSIs. In a marginal fraction, the specific type of SSI remained elusive, accounting for a mere 0.5% of the collective reported SSIs [5].

### 1.2. Risk Factors

In the relentless pursuit of averting surgical site infections (SSIs), a two-pronged approach unfolds, centering on the identification of risk factors that collectively fall into two distinctive categories: patient-related (intrinsic) and process/procedural-related (extrinsic) factors. These risk factors, whether modifiable or non-modifiable, constitute pivotal keystones in the campaign for infection prevention.

Patient-related risk factors include factors intrinsic to the patient’s health, such as underlying medical conditions or immunosuppression. Modifiable patient-related risk factors may include behaviors such as smoking, alcoholism, or obesity. Habits such as smoking, alcoholism, or obesity profoundly impact wound healing and immune function. For example, smoking impairs oxygen delivery to tissues, which is crucial for healing, thereby increasing the risk of infection [6]. Alcoholism can lead to malnutrition and weakened immune response, whereas obesity often complicates wound closure and increases the susceptibility of the tissue to infection [7].

Non-modifiable patient-related risk factors may include demographic factors such as age or gender [8]. Older patients often have reduced immune function and may have comorbidities that complicate surgery and recovery, increasing SSI risk. Sex-specific factors might influence the type and frequency of surgeries performed and, consequently, the associated SSI risk.

Beyond the individual, the surgical process itself becomes an intricate arena riddled with potential hazards. Process or procedural-related risk factors, extending from the extrinsic realm, encompass elements inherent to the surgical maneuver. Process- and procedure-related risk factors are extrinsic risk factors associated with the surgical process. These may include intraoperative risk factors such as the maintenance of asepsis or the duration of surgery. This panorama encompasses a multitude of variables, ranging from intraoperative factors such as aseptic practices to the duration of the surgical procedure. Encompassing a broader perspective, healthcare institutions bear the onus of extrinsic risk factors, spanning from ventilation standards to equipment sterilization protocols (Table 2). By meticulously attending to these external variables, a momentous stride is taken towards orchestrating a favorable surgical denouement and profoundly curtailing the incidence of surgical wound infections [9].

Additionally, healthcare institutions may be responsible for extrinsic risk factors such as poor ventilation or inadequate equipment sterilization. The adherence to infection control protocols, including preoperative antibiotic prophylaxis, can greatly influence SSI outcomes. Hospitals with strict adherence to these protocols generally report lower SSI rates. The quality of postoperative care, including wound care and monitoring for signs of infection, is vital. Early detection and treatment of SSIs can prevent more severe complications. Addressing these extrinsic risk factors can significantly improve the likelihood of a positive surgical outcome and decrease the incidence of surgical wound infections [9].

These risk factors do not operate in isolation. For example, a patient with obesity (intrinsic factor) undergoing lengthy abdominal surgery (extrinsic factor) in a facility with suboptimal sterilization practices (healthcare institution-related factor) is at a compounded risk for SSI.

Understanding the interplay between these factors is crucial for developing comprehensive strategies to reduce the incidence of SSIs. This includes personalized patient care plans that consider intrinsic factors and rigorous adherence to best practices in surgical procedures and facility management.

By addressing these risk factors, healthcare providers can significantly reduce the incidence of SSIs, leading to improved patient outcomes, shorter hospital stays, and reduced healthcare costs. This comprehensive understanding of the risk factors is essential for developing targeted preventive measures and enhancing the overall quality of surgical care.

### 1.3. Etiology

Generally, the panorama of surgical site infections (SSIs) in general surgery remains notably consistent [10,11,12]. Anchored in this temporal continuum is a constellation of microorganisms that orchestrate these infections, primarily populated by Gram-positive bacteria like coagulase-negative staphylococci (CNS), *Staphylococcus aureus*, and *Enterococcus faecalis* or *Enterococcus faecium*, alongside their Gram-negative counterparts including *Escherichia coli*, *Enterobacter cloacae* or *Enterobacter aerogenes*, *Klebsiella* spp., and *Pseudomonas aeruginosa*. Notably, while the tides of time have surged, the prominence of these microbial actors has endured, serving as the key protagonists in this recurrent tale.

Delving into the specifics, 10% of postoperative wound infections in general surgery trace their origins to methicillin-resistant *Staphylococcus aureus* (MRSA), a testament to the ongoing challenge posed by antimicrobial resistance. In contrast, the realm of multidrug-resistant Gram-negative (MRGN) bacteria has yet to make its presence felt, with no documented cases breaching this barrier.

While the microbial stage takes center spotlight, it is intriguing to note that yeasts, notably the *Candida* spp., assume a far more peripheral role as the culprits behind surgical site infections, accounting for a mere 3% of the instances. This nuanced realm is often accompanied by a chorus of concomitant risk factors, a symphony woven from elements like diabetes, chemotherapy, immunosuppression, and malnutrition, which collectively amplify the vulnerability to these infections. Also, it is worth acknowledging the sporadic role that viruses play in instigating surgical site infections [13,14]. These instances, while rare, cast a spotlight on the dynamic interplay between the biological and surgical realms, adding yet another layer to the intricate tapestry of infection causation.

### 1.4. Objectives

The primary objective of this systematic review was to ascertain the prevalence of surgical site infections (SSIs) across various surgical procedures. Additionally, this study aimed to identify and categorize the bacterial species most commonly associated with SSIs, thereby providing a clearer understanding of their etiological roles. Through this analysis, we seek to offer a nuanced perspective on the multifaceted factors contributing to SSIs, with the overarching goal of facilitating more effective management and mitigation strategies. By comprehensively exploring these dimensions, our endeavor was to enhance patient safety and the quality of surgical care, particularly in the context of evolving healthcare challenges and antimicrobial resistance.

## 2. Materials and Methods

### 2.1. Study Design and Selection Criteria

The findings of this review were articulated following the Preferred Reporting Items for Systematic Reviews and Meta-Analysis (PRISMA) statement guidelines, employing the PRISMA checklist to ensure robust reporting. The full trajectory of study screening, selection, and inclusion was comprehensively delineated with the aid of a flow diagram, enhancing the clarity and transparency of our review process. The current systematic review was registered in the PROSPERO registry for systematic review protocols under ID CRD42023446221. 

The field of surgery, including preoperative, intraoperative, and postoperative practices, has undergone significant changes in recent years owing to technological advancements and evolving medical standards. By focusing on the most recent decade, we aimed to capture data that reflect current surgical practices and the latest preventive measures against surgical site infections. Furthermore, the landscape of microbial pathogens responsible for SSIs and their antibiotic resistance patterns has shifted considerably over the last decade. Hospital environments, sterilization techniques, and patient management strategies have evolved rapidly. Recent studies are likely to provide insights that are directly applicable to current healthcare settings, making our findings more relevant to contemporary practitioners.

We performed literature research on the online databases PubMed, ScienceDirect, and Scopus over the last 10 years (2012–2022) using search strings such as “surgical site infections”, “wound infections”, and “surgical etiology”. All results were filtered for the English language, human patients, original work, and free access. Both adult and pediatric patients were included in this study. The initial search yielded a total of 409 results. Our inclusion criteria focused on epidemiological, cohort, transversal observational, and meta-analysis studies to ensure that we analyzed direct evidence of SSI prevalence, thereby excluding reviews, comments, and non-observational studies. 

**Figure 2:**
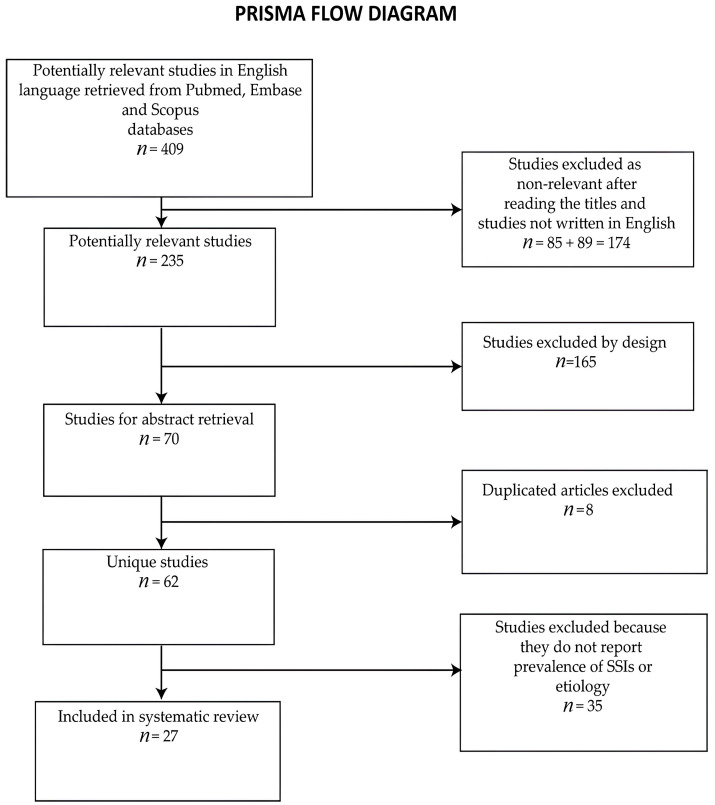
PRISMA flow diagram of the study selection process.

We excluded 89 studies that were not written in English. After excluding 28 duplicate studies, we read the titles and excluded 85 studies that were unrelated to the subjects. Therefore, 72 studies were selected for abstract retrieval. After reading the abstracts, we excluded 43 studies that did not report the prevalence rates of surgical site infections or did not enumerate the involved bacterial species. Finally, we selected 26 retrospective and prospective studies and 1 case control for inclusion in the review (Figure 2).

We extracted the relevant data from each study: title, authors, year, number of participants, SSI rate, involved bacteria, and antibiotic resistance profiles (Table 3).

### 2.2. Data Extraction

Each title and abstract were independently reviewed by two researchers (A.B. and D.D.V.) in line with our inclusion and exclusion criteria. Any discrepancy between the two researchers during the screening process was settled through conversation or the involvement of a third senior researcher (S.S.M. or F.G.H). The article was added to the entire read set if there was any lingering uncertainty. The following data items were collected from each study: title, authors, year, number of participants, SSI rate, involved bacteria, and antibiotic resistance profiles.

### 2.3. Quality Assessment

In our systematic review, we employed the National Heart, Lung, and Blood Institute (NHLBI) Study Quality Assessment Tools to evaluate the methodological quality of included studies [15]. This scoring system involves a checklist of criteria tailored to specific study designs (e.g., cohort, case-control, randomized controlled trials). Each criterion addresses aspects like study population, sample size, outcome measures, and statistical analysis.

For each study, investigators V.B.S. and M.I.S. independently assessed and scored these criteria. The scores were then used to categorize each study’s quality as “high”, “medium”, or “low”. This categorization helped us determine the reliability of each study’s findings and their contribution to our overall understanding of surgical site infection (SSI) prevalences.

In the context of our study, a higher score indicated a study with robust methodology and lower risk of bias, thus providing more reliable evidence. Conversely, lower scores indicated potential methodological weaknesses or biases. This scoring system was crucial in synthesizing the most valid and reliable evidence regarding SSI prevalences from the reviewed literature.

## 3. Results

The main study findings are presented in Table 3 and describe the type of surgery discussed in the study, the number of participants, the rate of infection, the etiological agents, and their frequency.

**Table 3:** Analyzed studies.

Authors	Type of Surgery	Period of Study, Region, Centers Involved	No. of Participants	SSI Rate	Etiology	Quality Score
Elliman et al., 2017 [11]	Cardiac surgery Orthopedic surgery Colorectal surgery Vascular surgery Hysterectomy	2008–2013 USA National Veterans Affairs cohort	70,101	3.5%	*S. aureus* MRSA—7.9% *S. aureus* MSSA—24.1% CNS—12% *Streptococcus* spp.—0% *Enterobacteriaceae*—40%	13
Shah et al., 2020 [12]	General surgery	2013–2018 India 1 hospital	55,553	1.% (0.97% in clean surgeries and 0.03% in contaminated surgeries)	*S. aureus*—12% *S. epidermidis*—7% *Streptococcus* spp.—0% *P. aeruginosa*—13% *K. pneumoniae*—17% *E. coli*—19%	11
Hou et al., 2020 [16]	Colorectal surgery	2015–2018 China 19 hospitals	3663	3.7% Superficial SSIs 2.1% Deep SSIs 0.7%	*S. aureus*—3.7% *S. epidermidis*—0% *Streptococcus* spp.—0% *P. aeruginosa*—6.00% *K. pneumoniae*—7.5% *E. coli*—40.3%	12
Panos et al., 2021 [17]	Colorectal surgery	2019–2021 Greece 1 hospital	133	21.8% Superficial SSIs 15% Deep SSIs 6% Organ space 0.8%	*S. aureus* 4% CNS- *Streptococcus* spp.— *P. aeruginosa*— *Klebsiella pneumoniae*—12% *E. coli*—44%	12
Du et al., 2019 [18]	Colorectal surgery	2015–2016 China 26 hospitals	5729	3.6% Radical resection of colon SSI 2.6% Superficial SSIs 0.9% Deep SSIs 0.6% Organ 1.1% Radical resection of rectum SSI 5.1% Superficial SSIs 2.3% Deep SSIs 1.1% Organ 1.7%	Radical resection of colon: *S. aureus*— *Streptococcus* spp.— *P. aeruginosa*—13.2% *Klebsiella pneumoniae*—7.9% *E. coli*—55.3% Radical resection of rectum: *S. aureus*— *Streptococcus* spp.— *Pseudomonas aeruginosa*—6.2% *Klebsiella pneumoniae*—6.2% *E. coli*—36.9%	12
Alkaaki et al., 2019 [19]	Abdominal surgery	2016 Saudi Arabia 1 hospital	337	16.3%	Gram-positive cocci—38% *P. aeruginosa*—14% *K. pneumoniae*—20% *E. coli*—52%	11
Benito et al., 2014 [20]	Knee/hip arthroplasty	2004–2010 Barcelona 1 hospital	2333 (knee/hip arthroplasties)	4.2%	*S. aureus* MRSA—28.6% *S. aureus* MSSA—6.8% CNS—24.2% *P. aeruginosa*—11.1%	12
Taherpour et al., 2021 [21]	Orthopedic surgery	2017–2018 Iran 6 hospitals	503	-	*E. coli*—11.1% *S. aureus*—23% CNS—8% *P. aeruginosa*—7% *Klebsiella* spp.—22% *E. coli*—8%	11
Slowik et al., 2020 [22]	Knee/Hip Arthroplasties	2012–2018 Poland 1 hospital	2340	1.6%	*S. aureus*—18.9% CNS—35.1% *Streptococcus* spp.— *P. aeruginosa*— *Klebsiella pneumoniae*—10.8% *E. coli*—2.7%	11
Meng et al., 2020 [23]	Foot and ankle surgery	2015–2018 China 1 hospital	1201	2.1% (1.3% superficial SSI; 0.8% deep SSI)	*S. aureus* MRSA—23.1% *S. aureus* MSSA—19.2% CNS—7.7% *Streptococcus* spp.— *P. aeruginosa*— *Klebsiella* spp.— *E. coli*—7.7%	11
Mathur et al., 2022 [24]	Orthopedic surgery	2018–2019 India 1 hospital	850	5.5%	*S. aureus*—36% CNS—2% *Streptococcus* pyogenes—4% *P. aeruginosa*—8% *Klebsiella pneumoniae*—10% *E. coli*—12%	12
Nagaya et al., 2017 [25]	Orthopedic surgery	1987–2012 Brazil 1 hospital	158	9.5%	*S. aureus*—12.5% CNS—6.2% *Streptococcus* spp.— *Pseudomonas aeruginosa* 18.7% *Klebsiella pneumoniae*— *E. coli*—	12
Maritati et al., 2022 [26]	Orthopedic surgery	2019–2020 Italy 1 hospital	760	3.3%	*S. aureus*—10% CNS—40% *Streptococcus* spp.— *Pseudomonas aeruginosa*—10% *Klebsiella pneumoniae* – *E. coli*—	11
Lu et al., 2019 [27]	Orthopedic surgery	2013–2017 China 3 hospitals	895	4% Deep SSIs 1.5% Superficial SSIs 2.5%	*S. aureus*—42.1% CNS—15.8% *Streptococcus* spp.— *Pseudomonas aeruginosa*—5.3% *Klebsiella pneumoniae*— *E. coli*—	11
Wang et al., 2018 [28]	Orthopedic surgery	2014–2017 China 1 hospital	725	9.7% Deep SSIs 2.9% Superficial SSIs 6.8%	*S. aureus*—19.7% *S. aureus* MRSA—2.1% CNS *Streptococcus* spp.— *Pseudomonas aeruginosa* 1.5% *Klebsiella pneumoniae*— *E. coli*—6.1%	11
Sun et al., 2018 [29]	Orthopedic surgery	2015–2016 China 3 hospitals	1511	4.4% Deep SSIs 1.3% Superficial SSIs 3.1%	MRSA—29.7% CNS *Streptococcus* spp.— *Pseudomonas aeruginosa* *Klebsiella pneumoniae*— *E. coli*—	11
Kahl et al., 2019 [30]	Cardiac surgery	2018 Spain 1 hospital	150	29.3%	*S. aureus*—8.8% CNS—8% *P. aeruginosa*—15.6% *Klebsiella* spp.—20.6% *E. coli*—4.2%	12
AlFawaz et al., 2022 [31]	Vascular surgery	2014–2019 Kuwait 1 hospital	391	14%	*S. aureus*—25.5% CNS— *Streptococcus* spp.— *P. aeruginosa*—34% *Klebsiella pneumoniae*— *E. coli*—13%	11
Banjanovic et al., 2022 [32]	Cardiac surgery	2015–2020 Bosnia 1 hospital	15	14.9%	MRSA—9% CNS— *Streptococcus* spp.— *P. aeruginosa*—4.5% *Klebsiella pneumoniae*—13% *E. coli*—4.5%	10
Zejnullahu et al., 2019 [33]	Obstetrics	2018 Kosovo 1 hospital	325	9.9%	*S. aureus*—28.1% CNS—6.3% *Streptococcus* spp.— *P. aeruginosa*—3.1% *Klebsiella* spp.—3.1% *E. coli*—9.4%	12
Gupta et al., 2021 [34]	Obstetrics surgery	2016 India 1 hospital	611	10.3% 66.7% superficial SSI; 27% deep SSI; 6.3% organ space	MSSA—42% MRSA—3% *Streptococcus* spp.— *P. aeruginosa*—5% *Klebsiella* pneumoniae—24% *E. coli*—13%	12
Zhang et al., 2022 [35]	Spinal surgery	2010–2020 China 1 hospital	521	1.8%	*S. aureus* *Streptococcus* spp.— *Pseudomonas aeruginosa* 10% *Klebsiella pneumoniae*— *E. coli* 6.7%	12
Pei et al., 2021 [36]	Spinal surgery	2016–2019 China 1 hospital	1269	3.4%	*S. aureus* MRSA—16% MRCNS—48% *Streptococcus* spp.— *Pseudomonas aeruginosa* *Klebsiella pneumoniae*—	11
Hamdeh et al., 2014 [37]	Neurosurgery	2010 Sweden	448	4.3%	ESBL—32% *E. coli* *S. aureus*—21.7% CNS—34.8%	12
Pereira et al., 2017 [38]	Neurosurgery	2011–2014 Spain 1 hospital	521	4.9%	*S. aureus*—23.1% *S. epidermidis*—23.1% *Streptococcus* spp.—11.5% *P. aeruginosa*—11.5% *K. pneumoniae*—11.5% *E. coli*—3.9%	11
Kolpa et al., 2019 [39]	Neurosurgery	2003- 2017 Poland 1 hospital	10,332	1.5%	*S. aureus*—49.7% CNS—3.2% *Streptococcus* spp.—1.9% *P. aeruginosa*—4.5% *Klebsiella* spp.—1.3% *E. coli*—5.7%	12
Morikane, 2018 [40]	Thoracic surgery	2012–2014 Japan Japan Nosocomial Infections Surveillance database	3538	4.1%	MRSA—19.2% CNS—16.4% *Streptococcus* spp.— *P. aeruginosa*— *Klebsiella* pneumoniae— *E. coli*—3.4%	12

MRSA—Methicillin-Resistant *Staphylococcus aureus*; MSSA—Methicillin-sensitive *Staphylococcus aureus*; CNS—Coagulase-Negative Staphylococci.

### 3.1. Colorectal Surgery

Two studies have investigated the incidence, risk factors, and microbiology of SSI after colorectal surgery (CRS). In both studies, *Escherichia coli* was identified as the most frequently isolated bacteria, and superficial infections were observed to occur more frequently than deep or organ infections [16,17].

A significant incidence of antibiotic resistance was found, with rates of carbapenem-resistant or extended-spectrum beta-lactamase-producing *Escherichia coli* and *Klebsiella* pneumonia at 50% (27 cases) and 30% (3 cases).

The two studies investigated a variety of SSI risk factors. Extrinsic risk factors, such as emergency surgery, a higher ASA score, contaminated or dirty wounds, prolonged operative times, and corticosteroid treatment, were identified as having a strong association with a higher rate of SSIs. In addition, intrinsic risk factors such as male gender, age over 60, body mass index (BMI) > 30 kg/m^2^, and diabetes were identified.

It was shown that the site of the tumor can influence the etiology of SSIs. In a study of 26 hospitals in China, Du et al. investigated the epidemiological distribution of SSIs in radical resections of the colon or rectum for malignancy [18]. The study reported an infection rate of 3.60%. Following the radical resection of rectal cancer, the incidence of SSI was 2.1 times greater than following the radical resection of colon carcinoma. 

In accordance with previous studies, the prevalence of infections was greater in superficial incisional SSIs than that of deep or organ space and *Escherichia coli* was identified as the most prevalent pathogen responsible for surgical site infections following radical colon cancer excision and rectal cancer. 

### 3.2. Abdominal Surgery

Alkaaki et al. conducted a prospective study to assess the incidence, bacteriology, and risk factors of surgical site infections in patients following abdominal surgery [19]. The study evaluated a total of 337 patients who underwent abdominal surgery over a one-year period. Of these, 55 patients developed SSIs, representing an overall incidence rate of 16.3%, of which 9% were deep. Among risk factors, the open surgical method, emergency surgery, and prolonged operation length were the most significant extrinsic risk factors, and the masculine gender was the most important non-modifiable intrinsic risk factor.

*Escherichia coli* strains that produce an extended-spectrum beta-lactamase were the most often isolated bacteria, followed by *Enterococcus* spp. This result is in accordance with other research on colorectal surgery, which also identified *Escherichia coli* as a pathogen that was commonly involved.

### 3.3. Orthopedic Surgery

In a study conducted at the Hospital de la Santa Creu I Sant Pau in Barcelona, Benito et al. examined the incidence of surgical site infections (SSIs) following a total hip and knee arthroplasty [20]. They analyzed 2333 patients with knee/hip arthroplasties performed between 2004 and 2010. During the study period, the yearly incidence of SSIs did not change considerably (4.20%). In the same study, staphylococci were the most prevalent cause of monomicrobial infections (62.2%). A total of 28.6% of *S. aureus* isolates showed resistance to methicillin, while GNB and *Enterococcus* spp. Were the most common pathogens isolated from polymicrobial infections.

Another study on 503 patients found an infection rate of 39.60% in open reduction and internal fixation, 5.55% during total hip arthroplasty, and 9.40% in total knee arthroplasty. Most SSIs were detected after discharge from the hospital [21].

In a study on 2340 patients conducted in Poland, CNS (35.10%) and *S. aureus* (18.90%) were the main causes of hip and knee infections [22]. All strains of *S. aureus* were susceptible to methicillin, vancomycin, and linezolid. One case of *Acinetobacter baumannii* was found in non-fermenting rods. This case was 100% resistant to cefepime, cefotaxime, ceftazidime, and ciprofloxacin, but it was still sensitive to carbapenem. However, during the study period, the proportion of SSIs due to GNB and polymicrobial infections significantly increased. In addition, a reduction in CNS proportion was also observed. During this time period, there was no significant change in the annual incidence of SSIs.

A study of 1201 patients revealed that there are five distinct factors that exhibit an independent association with SSIs [23]. These factors include an extended preoperative stay, the use of an allograft or bone substitute, an elevated fasting blood glucose level, a lower albumin level, and an abnormal neutrophil count. Risk factors that predict the development of SSIs are total leucocyte counts higher than 7860/L and neutrophil counts higher than 5185/L (with a high degree of accuracy) [23,26]. Additional risk factors include open fracture, obesity, smoking, diabetes mellitus [27], ASA class 3 or higher, and an intraoperative temperature of 36.0 °C [28].

### 3.4. Cardiovascular and Cardiac Surgery

The incidences, risk factors, and microbiology of surgical site infections following cardiovascular surgery have been analyzed in three studies. 

Banjanovic et al. conducted a study on 2340 patients and observed that 37% of patients had two risk factors for deep sternal wound infection (25% were diabetic patients and 3% were overweight) [32]. In regard to etiology, *Enterococcus faecalis* was the most prevalent pathogen (27%), followed by *Klebsiella pneumoniae* (13%), *Proteus mirabilis* (9%), and *Serratia marcescens* (9%). In contrast, Morikane et al. observed that MRSA and CNS were the predominant pathogens responsible for surgical site infections [40].

Kahl et al. focused on the prevalence of comorbidities, types of surgical procedures, and microbiology findings related to SSI in a cohort of patients, in which the most prevalent surgical procedure was myocardium revascularization surgery. The total incidence was 29.3%, and the most prevalent comorbidities were systemic arterial hypertension (74.7%), diabetes mellitus (44.7%), and dyslipidemia (40.0%). Myocardium revascularization surgery was the most prevalent surgical procedure (79.3%) [30].

### 3.5. Obstetrics Surgery

Two studies focused on the incidence of SSIs following caesarean sections [33,34]. In 55.3% of the cases, the identified pathogens were Gram-negative bacteria. *Klebsiella pneumoniae* was the most prevalent Gram-negative bacterium, followed by *Escherichia coli* and *Acinetobacter* spp. Furthermore, *S. aureus* represented 28.1% of all identified microorganisms, which makes it a significant pathogen.

Gram-negative bacteria were susceptible to aminoglycosides, fluoroquinolones, and carbapenems but resistant to cephalosporins and amoxicillin-clavulanate. Gram-positive bacteria, on the other hand, were sensitive to aminoglycosides, fluoroquinolones, clindamycin, and vancomycin but resistant to amoxicillin-clavulanate and erythromycin.

The identified risk factors for SSIs include inadequate pre-operative antibiotic prophylaxis, intra-operative blood transfusions, and comorbidities such as cardiovascular diseases, hypothyroidism, and chronic liver and renal disease.

### 3.6. Neurosurgery

Pereira et al. and Hamdeh et al. aimed to examine the prevalence, impact, and potential risk factors of surgical site infections in neurosurgery [37,38]. The rate of SSIs was 4.85%, with a total of 26 infections. These infections were classified as 65.38% organ space infections, 30.77% deep infections, and 7.69% superficial infections. *Staphylococcus epidermidis* was the most frequently isolated microorganism, accounting for 23.08% and 34.8% of cases, respectively.

Patients who received appropriate preoperative antibiotic prophylaxis had a significantly lower incidence of SSIs (4.43%) compared to those who received inappropriate or no antibiotic prophylaxis (11.23%). Additional risk factors are associated with SSIs, including meningiomas, longer operation times, craniotomies, and the use of dural substitutes.

In another study conducted by Kolpa et al. SSIs accounted for 33% of all HAI incidences during the study period, with *S. aureus* being the most prevalent causative agent (35.9%), followed by CNS (28.9%). The most significant HAI incidence rates were observed in ventricular shunt implantation surgeries (77 cases, 18.6%) and craniotomies (235 cases, 8.0%) [39].

### 3.7. Spinal Surgery

The incidence, characteristics, and risk factors associated with surgical site infections following posterior thoracolumbar and lumbar instrumentation were analyzed in a study elaborated by Zhang et al. [35]. They observed that patients with lumbar spinal stenosis had a substantially higher infection rate than patients with scoliosis or kyphosis. A total of 521 (1.8%) patients were diagnosed with SSIs among the 27,881 procedures extracted from the databases, representing an average SSI rate of 1.8%.

Another study conducted by Pei et al. on 1269 patients found an incidence rate of 3.4% in posterior lumbar interbody fusion and instrumentation in lumbar degenerative disease patients [36]. MSSA (43.4%) was the most frequently identified pathogen, while methicillin-resistant coagulase-negative *Staphylococcus* was the most prevalent of the identified drug-resistant genotypes.

## 4. Discussion

In our systematic review, we identified a constellation of risk factors associated with an increased incidence of surgical site infections (SSIs). Predominantly, patient-related factors such as comorbidities, advanced age, higher BMI, and negative behaviors like addictions emerged as significant contributors to SSI risk. Notably, conditions such as diabetes and immunosuppression were frequently cited in the reviewed articles as exacerbating factors. 

The procedural characteristics, including the duration and complexity of surgeries, also played a crucial role. This finding aligns with the existing literature suggesting that prolonged surgical time increases the opportunity for pathogen entry, complicating postoperative wound management. 

In cardiovascular surgery, the etiology of surgical site infections has been diverse; *Enterococcus faecalis*, *Klebsiella pneumoniae*, and MRSA are the most frequently implicated agents. Comorbidities such as systemic arterial hypertension and diabetes mellitus, which are prevalent in the population, have been the most important identified factors in relation to SSI incidence, highlighting the need to optimize patients’ overall health in order to re-duce the risk of postoperative infections in this type of surgery in regard to their chronic pathological status. 

In colorectal and abdominal surgery, *Escherichia coli* is the bacterium most frequently isolated from SSIs. Additionally, there could also be other pathogens involved, such as *Enterococcus* spp.

These findings emphasize the significance of prevention strategies that target *Escherichia coli* and the need to implement successful antimicrobial management programs to fight the high prevalence of antibiotic resistance, particularly in carbapenem-resistant or extended-spectrum beta-lactamase-producing strains.

Furthermore, the duration of pre-operative hospital stays was identified as a healthcare-related risk factor, possibly due to prolonged exposure to hospital-acquired pathogens. 

Our findings underscore the need for a multifaceted approach to SSI prevention, integrating rigorous pre-operative assessments and tailored postoperative care, especially for high-risk patient groups. However, the heterogeneity of the included studies and potential biases highlight the necessity for future focused research. Prospective studies investigating the impact of specific interventions on reducing SSIs in patients with these risk factors would be invaluable in enhancing patient outcomes and surgical safety.

In this type of surgery, previous studies demonstrate that minimally invasive surgery is associated with a reduction in SSI incidence [41,42]. 

It was shown that SSIs are independently correlated with multiple distinct factors. These factors include hypertension; coronary artery disease; pulmonary disorders; ASA III and above; preoperative stay; longer and/or more complicated surgical procedures; and patient conditions such as diabetes mellitus, renal insufficiency, long-term prophylactic antibiotic use, and decreased blood lymphocyte counts [36]. 

### Limitations

This systematic review exploring the origins of surgical site infections is not without its drawbacks. We limited our search for qualifying studies to only a few databases, PuMed, Embase, and Scopus, which may have led to the omission of pertinent studies from other databases. There is a chance that bias may have influenced the selection of incorporated studies that are mostly observational studies. The possibility of publication bias, wherein studies showing null results are published less often, cannot be ignored. Our decision to limit included studies to those published within the last 10 years may have excluded valuable historical data. While this approach ensured relevance to current medical practices, it may have overlooked the evolution of SSI trends and long-term shifts in risk factors.

This systematic review has focused exclusively on exploring surgical site infections through the lens of open access articles. While this approach has enabled a broad and accessible overview of available research in this domain, it inherently presents certain limitations. Notably, the exclusion of non-open-access articles might have led to a potential selection bias, omitting valuable insights and findings that are only available in publications behind paywalls. Consequently, this might have restricted the comprehensiveness of our analysis as some relevant studies could have been overlooked.

By focusing exclusively on epidemiologic, cohort, transversal observational, and meta-analysis studies, we aimed for direct evidence on SSI prevalences. This exclusion of reviews, comments, and other non-observational studies means that broader, qualitative insights into SSIs may have been overlooked.

We employed the NHLBI’s Study Quality Assessment Tools for evaluating study quality. Although this method is robust, it inherently relies on subjective judgment to some extent. Different assessors might interpret criteria slightly differently, which could affect the scoring and subsequent interpretation of study quality.

Moreover, significant variations in the methods and definitions of infection employed across different studies also pose limitations, such as sample size and the type of surgery. In addition, certain variables that were either not reported or reported insufficiently might have affected the findings. 

Finally, the variety in infection etiology across different regions might not have been adequately depicted due to geographic bias in the chosen studies. Another limitation of the study was that it was challenging to compare studies because of differences in study characteristics, variable definitions, procedures included, and study quality.

## 5. Conclusions

This systematic review revealed a variety of risk factors for SSIs as well as the most prevalent microorganisms responsible for these infections in different types of surgeries. This systematic review has revealed a variety of risk factors for SSIs, as well as the most prevalent microorganisms responsible for these infections in different types of surgeries. A comprehensive understanding of the etiological profile of SSIs is imperative for optimizing therapeutic strategies aimed at reducing their incidence. In particular, cardiovascular and colorectal procedures have the highest SSI rates, with *Enterococcus faecalis*, *Klebsiella pneumoniae*, and methicillin-resistant *Staphylococcus aureus* (MRSA) emerging as the most prevalent pathogens in cardiovascular procedures, while *Escherichia coli* has played an important role in colorectal surgery. Particularly, cardiovascular and colorectal procedures have the highest SSI rates, with *Enterococcus faecalis*, *Klebsiella pneumoniae*, and methicillin-resistant *Staphylococcus aureus* (MRSA) emerging as the most prevalent pathogens in cardiovascular procedures, while *Escherichia coli* has exhibited an important role in colorectal surgery.

Our findings provide significant information for surgeons, anesthesiologists, infectious disease doctors, and microbiologists to initiate a prospective trial to study the role of risk factors for SSIs in surgical patients. Our findings provide significant information for surgeons, anesthesiologists, infectious disease doctors, and microbiologists to initiate a prospective trial to study the role of risk factors in SSIs in surgical patients.

## Figures and Tables

**Table 1:** CDC classification of surgical wound infections.

Classification	Definition	Criteria
Superficial Incisional SSI	Infection occurs within 30 days after the operation and involves only skin and subcutaneous tissue of the incision.	Purulent drainage, with or without laboratory confirmation, from the superficial incision.
Deep Incisional SSI	Infection occurs within 30 days after the operation and involves deep soft tissues, such as fascial and muscle layers.	Purulent drainage from the deep incision but not from the organ/space component of the surgical site.
Organ/Space SSI	Infection occurs within 30 days after the operation and involves any part of the anatomy (e.g., organs or spaces) other than the incision, which was opened or manipulated during the operation.	Infection involving any part of the anatomy (e.g., organs or spaces) other than the incision, which was opened or manipulated during the operation.

**Table 2:** Common risk factors for surgical site infections (SSIs).

Surgical Procedure	Common Risk Factors
Orthopedic Surgery	Prolonged surgery duration, foreign material (e.g., implants), inadequate sterilization
Cardiac Surgery	Use of heart–lung machine, lengthy operations, blood transfusions
Abdominal Surgery	Emergency procedures, contamination of the surgical site, prolonged preoperative stay
Transplant Surgery	Immunosuppressive therapy, allograft contamination, technical issues
Neurosurgery	Shaving of the surgical site, dural exposure, implant use

## Data Availability

Data are available upon request from the correspondence author.
